# A new phantom and empirical formula for apparent diffusion coefficient measurement by a 3 Tesla magnetic resonance imaging scanner

**DOI:** 10.3892/ol.2014.2187

**Published:** 2014-05-28

**Authors:** MARINA HARA, MASAHIRO KURODA, YUICHI OHMURA, HIDENOBU MATSUZAKI, TOMOKI KOBAYASHI, JUN MURAKAMI, KAZUNORI KATASHIMA, MASAKAZU ASHIDA, SEIICHIRO OHNO, JUN-ICHI ASAUMI

**Affiliations:** 1 Department of Oral and Maxillofacial Radiology, Graduate School of Medicine, Dentistry and Pharmaceutical Sciences, Okayama University, Okayama 700-8558, Japan; 2 Department of Radiological Technology, Graduate School of Health Sciences, Okayama University, Okayama 700-8558, Japan; 3 Department of Oral Diagnosis and Dentomaxillofacial Radiology, Okayama University, Okayama 700-8558, Japan; 4 Central Division of Radiology, Okayama University Hospital, Okayama University, Okayama 700-8558, Japan

**Keywords:** sucrose, phantom, apparent diffusion coefficient value, diffusion-weighted imaging, magnetic resonance imaging, 3 Tesla

## Abstract

The aim of this study was to create a new phantom for a 3 Tesla (3T) magnetic resonance imaging (MRI) device for the calculation of the apparent diffusion coefficient (ADC) using diffusion-weighted imaging (DWI), and to mimic the ADC values of normal and tumor tissues at various temperatures, including the physiological body temperature of 37°C. The phantom was produced using several concentrations of sucrose from 0 to 1.2 M, and the DWI was performed using various phantom temperatures. The accurate ADC values were calculated using the DWIs of the phantoms, and an empirical formula was developed to calculate the ADC values of the phantoms from an arbitrary sucrose concentration and arbitrary phantom temperature. The empirical formula was able to produce ADC values ranging between 0.33 and 3.02×10^−3^ mm^2^/sec, which covered the range of ADC values of the human body that have been measured clinically by 3T MRI in previous studies. The phantom and empirical formula developed in this study may be available to mimic the ADC values of the clinical human lesion by 3T MRI.

## Introduction

Diffusion-weighted magnetic resonance imaging (MRI) has been increasingly performed for clinical purposes, including the detection of tumors and cerebrovascular diseases. The apparent diffusion coefficient (ADC) value, which is calculated based on diffusion-weighted imaging (DWI) using several b values, is useful for discriminating whether the lesion is benign or malignant and determining the therapeutic effect of a tumor. Recently, popularized 3 Tesla (3T) MRI devices have shown a performance advantage when calculating accurate ADC values. Several clinical studies have revealed that ADC values from 3T MRI have the diagnostic value as a quantitative parameter (1–8). However, to the best of our knowledge, there are no reports of an ADC phantom for 3T MRI. With regard to ADC phantoms for 1.5T MRI, Tamura *et al* (9) reported a phantom that used gelatin and sucrose. While Matsuya *et al* (10) reported a phantom using polyethylene glycol for 1.5T MRI, and created empirical formulas to calculate polyethylene glycol concentration, which provide arbitrary ADC values at any temperature measurement. In principle, the ADC value of a phantom differs due to its temperature. In the present study, an ADC phantom was developed using sucrose for 3T MRI, which produces arbitrary ADC values due to a range of phantom temperatures (28–39°C), which includes the physiological body temperature. This is the first temperature-controlled ADC phantom for 3T MRI, which mimics the ADC values of the normal and tumor tissues of the human body. In addition, the developed empirical formula enables the calculation of a sucrose concentration that provides arbitrary ADC values at any phantom temperature.

## Materials and methods

### Sucrose phantoms

**Figure 1:**
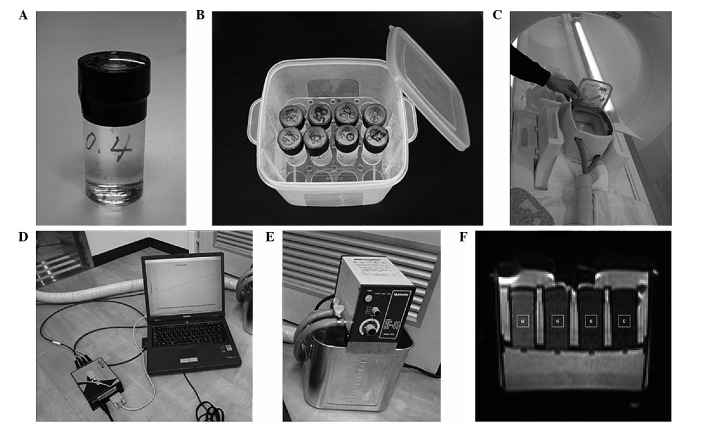
Phantom and methods used for the experiments. (A) Sucrose phantom in its case and (B) case container. Up to 16 sucrose phantoms could be placed into this container filled with 0.9 M sucrose solutions containing 0.03% (w/w) NaN_3_. (C) The heating box made of Styrofoam, which encloses the phantom case container. The container could be heated in the gantry of a magnetic resonance imaging scanner via a tube that was connected to a (D) circulating temperature-regulated water bath. (E) The optical fiber thermometer for temperature monitoring, which was placed into the phantoms. (F) The region of interest was 7.27 mm^2^ at the position of the thermometer on each diffusion-weighted image.

To create the sucrose phantoms, sucrose (S0389-500G; Sigma-Aldrich, St. Louis, MO, USA), NaN_3_ (28-1789-5; Sigma-Aldrich, Tokyo, Japan), as an antiseptic, and distilled water were heated and stirred until dissolved. The solution was cooled and the final concentrations of sucrose and NaN_3_ were adjusted to 0.2, 0.4, 0.6, 0.8, 1.0 and 1.2 M, and 0.03% (w/w), respectively. These solutions were then filled into phantom cases (No1-4628-11; As One Co., Osaka, Japan; Fig. 1A*)* as sucrose phantoms.

### Preparation for the MRI of sucrose phantoms

Sucrose phantoms were placed into a container filled with 0.9 M sucrose solution and 0.03% (w/w) NaN_3_. The container was able to hold a maximum of 16 phantoms (Fig. 1B).

### Heating system

The phantom case container was enclosed in a heating box (Fig. 1C*)* made of Styrofoam that was produced in-house (Department of Radiological Technology, Graduate School of Health Sciences, Okayama University, Okayama, Japan). The container was heated in the gantry of an MRI scanner via a tube that was connected to a circulating temperature-regulated water bath (Thermo-Mate BF-41; Yamato Scientific Co., Ltd., Tokyo, Japan; Fig. 1D), to maintain the desired phantom temperature during the MRI.

### Real-time phantom temperature monitoring

Optical fiber thermometers (Fluoroptic™ thermometer m600; Luxtron Co., Mountain View, CA, USA; Fig. 1E) were placed into the phantoms. The phantom temperature was monitored every 30 sec during the MRI to ensure a constant temperature.

### MRI

A clinical 3T MRI unit (Magnetom Skyra; Siemens, Erlangen, Germany) with a head coil was used for the MRI. DW images were acquired by a three-scan trace, in the phase-encoding, readout and slice-selective directions, via a single-shot echo-planar imaging sequence. The scan parameters were set as follows: 8,000 msec of relation time; 100 msec of echo time; 220×220-mm field of view; 160×112 matrix; b values of 0, 300, 600, 900, 1200, 1500, 1800, 2100, 2400, 2700 and 3000 sec/mm^2^; a thickness of 5 mm; one excitation number; 26.2-msec diffusion gradient pulse duration (δ); and 47.1-msec diffusion time (Δ), which was the interval between the onset of the diffusion gradient pulses. Each DW image of a maximum of four phantoms was obtained at each ~1°C interval to cover the physiological body temperature within the range of 28–39°C.

### Accurate measurement of ADC values

The region of interest (ROI; Fig. 1F) was 7.27 mm^2^ at the position of the thermometer on each phantom DW image. The average signal intensity in each ROI was obtained using Image-J software (National Institutes of Health, Bethesda, MD, USA). The logarithms of these signal intensities were plotted as a function of the 11 b values of 0, 300, 600, 900, 1200, 1500, 1800, 2100, 2400, 2700 and 3000 sec/mm^2^. The slope of the regression line, which is defined as the ADC value, and its R^2^ value were obtained by the least-squares method. The 10 sets of ADC values and their R^2^ values were obtained for each set of data, from 11 DW images using 11 b values to two DW images using two b values, in order of decreasing b value. We used 10 sets of DW images using the following combination of b values; 0, 300, 600, 900, 1200, 1500, 1800, 2100, 2400, 2700 and 3000; 0, 300, 600, 900, 1200, 1500, 1800, 2100, 2400 and 2700; 0, 300, 600, 900, 1200, 1500, 1800, 2100 and 2400; 0, 300, 600, 900, 1200, 1500, 1800 and 2100; 0, 300, 600, 900, 1200, 1500 and 1800 / 0, 300, 600, 900, 1200 and 1500; 0, 300, 600, 900 and 1200; 0, 300, 600 and 900; 0, 300 and 600; 0 and 300. When the R^2^ values exceeded 0.99 according to a decrease in b value, the ADC values from its set of b values was determined to be accurate; specifically, the b value used was within the range that the signal intensities remained above the noise, and where the slope of the logarithms of the signal intensities versus b values became linear. These accurate ADC values were used to create the following empirical formula.

### Empirical formula for calculating phantom ADC values

ADC values of the phantoms were plotted as a function of the temperature from 28–39°C at 1°C intervals for each sucrose concentration of 0, 0.2, 0.4, 0.6, 0.8, 1.0 and 1.2 M. The linear equations were determined for each sucrose concentration based on a first-order approximation of the correlation between the ADC values and the phantom temperature. The first-order coefficients and intercepts of the seven linear equations were also plotted as a function of the sucrose concentrations. Subsequently, two formulas were created; one based on the fourth-order approximation of the correlation between the first-order coefficients and sucrose concentrations, with the other based on the fourth-order approximation of the correlation between the intercepts and sucrose concentrations. Using these two formulas, an empirical formula was developed for calculating ADC values of phantoms that were made of arbitrary sucrose concentrations at arbitrary phantom temperatures.

### Validation of the accuracy of the empirical formula

To validate the accuracy of the empirical formula, new phantoms were produced using sucrose concentrations of 0.2, 0.4, 0.6, 0.8, 1.0 and 1.2 M. Three phantoms were made of each concentration and all sucrose concentrations were used three times independently. The mean ADC values were obtained at each concentration. The ADC values of these verification phantoms were measured at phantom temperatures ranging from 28–39°C at 1°C intervals. The experimental mean ADC values of these verification phantoms were compared with the ADC values calculated using the empirical formula by substituting the sucrose concentrations and phantom temperatures at measurement. The correlation between the ADC values calculated using the empirical formula and the range of the standard deviations (SDs) of the experimental ADC values of the verification phantoms were then validated.

## Results

### Calculation accuracy of ADC values

**Figure 2:**
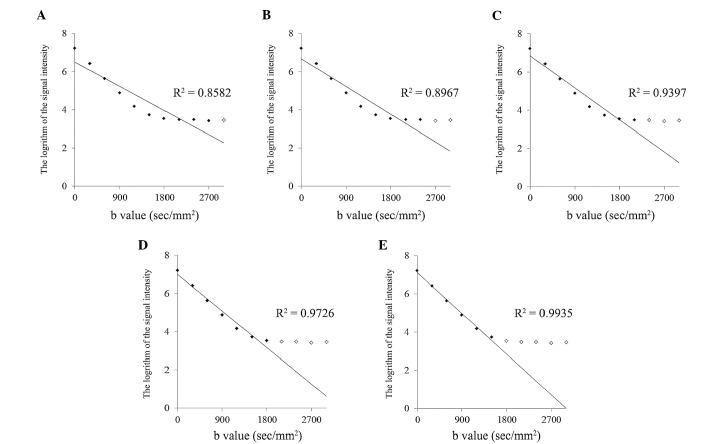
Calculation of apparent diffusion coefficient values of a 0.2 M phantom at 37.09°C. The vertical axis indicates the logarithm of the signal intensity of the regions of interest in the diffusion-weighted image of the phantom. The horizontal axis indicates b values. ‘♦’ represents the data that were used for the least-squares method to obtain the regression line and the R^2^ value. ‘⋄’ represents the data that were not used for the least-squares method to obtain the regression line and the R^2^ value.

**Figure 3:**
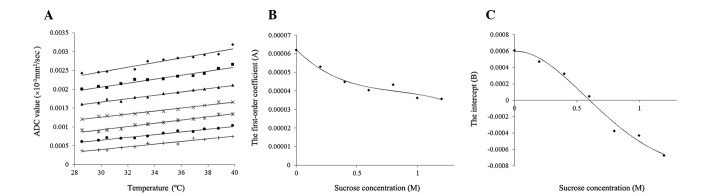
The ADC values of the phantoms and the development of an empirical formula to calculate the ADC values. (A) The change of ADC values by temperature. The vertical axis indicates the ADC values and the horizontal axis indicates the phantom temperature. Sucrose phantom concentrations of ⋄, 0; ■, 0.2; ▲, 0.4; ×, 0.6; *, 0.8; ●, 1.0; and +, 1.2 M. Each straight line indicates a first-order approximation of the correlation between the ADC values and the phantom temperature for each sucrose concentration. The (B) first-order coefficients and (C) intercepts of these linear equations are plotted. Each R^2^ value for the first-order approximation was within the range of 0.9379–0.9801. (B) The correlations between the sucrose concentrations and the first-order coefficients of linear equations from the first-order approximation. Black diamonds indicate first-order coefficients, while the curved line indicates the fourth-order approximation, with R^2^=0.9638. (C) The correlations between sucrose concentrations and the intercepts of the linear equations from the first-order approximation. Black diamonds indicate intercepts, while the curved line indicates the fourth-order approximation, with R^2^=0.9862. ADC, apparent diffusion coefficient.

For each concentration and temperature of the sucrose phantoms, the ADC values were calculated. The 10 sets of ADC values and their R^2^ values were obtained by the least-squares method for each set of data from 11 DW images using 11 b values to two DW images using two b values in order of decreasing b value. As an example, Fig. 2 indicates the procedure to calculate the ADC value of a 0.2 M phantom at a temperature of 37.09°C. Among 10 sets of ADC values and their R^2^ values, when the maximum b value decreased to 1,500 sec/mm^2^ (Fig. 2E), the R^2^ value obtained for the set of data from six DW images using six b values exceeded 0.99 to become 0.9935. According to the slope calculation using this set, the ADC value of the 0.2 M phantom became 3.72×10^−3^, which was confirmed to be accurate. Finally, the ADC values were selected for all concentrations and temperatures, as shown in Fig. 3A.

### Change in the ADC value of sucrose phantoms by temperature

The ADC values of the 0, 0.2, 0.4, 0.6, 0.8, 1.0 and 1.2 M phantoms are plotted in Fig. 3A as a function of temperature. The ADC values of the phantoms of each sucrose concentration increased with increasing phantom temperature. The increasing rate of the ADC value per 1°C increased as the sucrose concentration decreased.

### Development of an empirical formula to calculate ADC values

Seven linear equations were developed based on a first-order approximation of the correlation between the ADC values and phantom temperature (t) for each sucrose concentration (s), as shown in Fig. 3A. The values of these R^2^ were within the range of 0.9379–0.9801. The first-order coefficients (A) and intercepts (B) of the seven linear equations were plotted as a function of sucrose concentrations (s), as shown in Fig. 3B and C, respectively. Each formula was developed based on a fourth-order approximation of the correlation between the first-order coefficients or intercepts and sucrose concentrations. The R^2^ values were 0.9638 and 0.9862, respectively. Using these relational formulas, an empirical formula was developed for calculating the ADC values of phantoms consisting of an arbitrary sucrose concentration (s) at arbitrary phantom temperature (t), as follows: ADC value (x10^−3^ mm^2^/sec) = At + B, where A = a_1_s^4^ − a_2_s^3^ + a_3_s^2^ − a_4_s + a_5_ (a_1_=8.96519842127907×10^−7^, a_2_=2.94479295800953×10^−5^, a_3_=6.94789261608819×10^−5^, a_4_=6.5038339758676×10^−5^ and a_5_=6.22597789270809×10^−5^) and B = −b_1_s^4^ + b_2_s^3^ −b_3_s^2^ + b_4_s + b_5_ (b_1_=5.75284527700504×10^−4^, b_2_=2.48741270074326×10^−3^, b_3_=3.12590711150129×10^−3^, b_4_=1.19937338765919×10^−4^ and b_5_=5.94518521028771×10^−4^).

### Validation of the accuracy of the empirical formula

**Figure 4:**
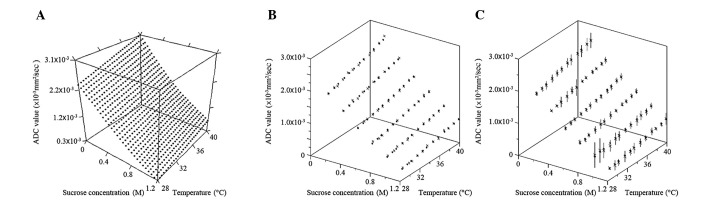
Calculated ADC values and validation of the accuracy using the empirical formula. The vertical axis indicates the ADC values and the horizontal axes indicate sucrose concentration and phantom temperature, respectively. Black cubes in each figure indicate the ADC values that were calculated from the empirical formula. (A) The ADC values which were calculated from the empirical formula. (B) The correlation between the predetermined and calculated ADC values using the empirical formula. The crosses (×) indicate the ADC values used to make the empirical formula. (C) The correlation between the ADC values measured using verification phantoms and the ADC values calculated using the empirical formula. The crosses (×) and vertical lines indicate the mean ± three standard deviations of the ADC values measured using verification phantoms. ADC, apparent diffusion coefficient.

Fig. 4A indicates the calculated ADC values using the empirical formula shown as the three-dimensional graph with the correlation among ADC values, sucrose concentration and phantom temperature. The ADC values decreased according to an increase in sucrose concentration and decrease in phantom temperature. Fig. 4B indicates the correlation between the ADC values, which have been used to make the empirical formula, and the ADC values calculated using the empirical formula. The formula appears to mimic well all the ADC values that were initially used to create it. Fig. 4C indicates the correlation between the ADC values measured using the verification phantoms and the ADC values calculated using the empirical formula. In total, 66.67% of the calculated ADC values were less than one SD away from the mean of the measured ADC values of verification phantoms; 97.22% of the calculated ADC values were less than two SDs away from the mean; and 100% of calculated ADC values were less than three SDs away from the mean.

## Discussion

To the best of our knowledge, this is the first study to report ADC phantoms for DW images with 3T MRI. ADC phantoms were produced for 3T MRI using sucrose, and an empirical formula was developed to calculate ADC values between 0.33–3.02×10^−3^ at arbitrary sucrose concentrations between 0–1.2 M and arbitrary phantom temperatures between 28–39°C, including the physiological temperature of 37°C to mimic the normal and tumor tissue of the human body.

Sucrose, a large molecule with the formula of C_12_H_22_O_11_, is a safe and inexpensive material, with a concentration that can be easily controlled. The diffusion coefficient of the material (D) was associated with the temperature (t), the viscosity of the medium (η), and the radius of the diffusion molecule (r) using the Stokes-Einstein equation (11): D = kt/6πηr, where k is the Boltzmann constant (1.3805×10^−23^ J K^−1^). Therefore, sucrose with a large molecular size of 0.9 nm in diameter was selected as the material for the phantoms to decrease the ADC values (12).

According to the Stokes-Einstein equation, ADC values are affected by the temperature of the objects in question. As the ADC values used in clinical MRI diagnosis are measured for the human body at 37°C, ADC phantoms that mimic human body tissue should be comparable. Sasaki *et al* (13) measured the ADC values of bio-phantoms using human Burkitt’s lymphoma cells at 37°C; however, the majority of *in vitro* studies have performed the ADC measurement at a lower temperature (14–16). Tamura *et al* (9) reported an ADC phantom using 10–50% (wt/wt) sucrose for 1.5T MRI, which covers the range of ADC values between 0.2 and 1.8×10^−3^ mm^2^/sec for temperatures between 6 and 20°C. In the pre-examination of the present study, the ADC values were measured at temperatures between 6–39°C. The R^2^ values of the first-order approximation of the correlation between the ADC values and phantom temperature were low for phantoms of high sucrose concentration at temperatures of <27°C. Therefore, the temperature range of 28–39°C was used to create the empirical formula.

This empirical formula covered ADC values from 0.672.47×10^−3^ mm^2^/sec at a physiological temperature of 37°C. The ADC values of the phantoms almost covered the ADC values of the normal and tumor tissues of the human body that are measured clinically by 3T MRI, as summarized in Table I (1,3,5–8,17–19). Table I indicates the sucrose concentration of the ADC phantoms at 37°C, which mimic each tissue of the human body using the empirical formula.

One limitation of this study was that the sucrose phantoms produced ADC values due to changes in free diffusion alone. The actual *in vivo* diffusion in the human body is affected not only by the change of free diffusion, but also various factors, including perfusion and the change of restricted diffusion, due to cellular membrane structures and cell density (20–26). This new ADC phantom and empirical formula for 3T MRI has the potential to be used in a number of applications.

## Figures and Tables

**Table I:** Sucrose concentration mimicking ADC values of human body.

Regions (ref)	Mean ADC values, ×10^−3^ mm^2^/sec	Sucrose concentration, M
Lesions		
Brain		
Lymphoma (6)	0.62^b^	~1.2
Head and neck		
Squamous cell carcinoma (1)	1.10	0.86
Thyroid gland		
Malignant tumor (19)	0.81^c^	1.07
Benign tumor (19)	1.55^c^	0.61
Pancreas		
Neoplastic cystic lesion (7)	2.60^b^	0.13
Mucinous cystic lesion (7)	2.60^b^	0.13
Uterine cervix		
Malignant tumor (3)	0.88^b^	1.02
Ovary		
Malignant tumor (8)	1.04^a^	0.91
Benign tumor (8)	1.15^a^	0.84
Prostate		
Peripheral zone tissue		
Malignant tumor (17)	0.85^d^	1.04
Benign tumor (17)	1.17^d^	0.82
Transition zone tissue		
Malignant tumor (17)	0.84^d^	1.05
Benign tumor (17)	1.08^d^	0.88
Normal tissues		
Brain		
White matter (18)	0.76^b^	1.11
Gray matter (18)	0.78^b^	1.10
Muscle		
Gluteus (3)	1.24^a^	0.78
Prostate		
Central gland (17)	1.19^d^	0.81
Peripheral gland (17)	1.54^d^	0.61
Tyroid tissue (19)	1.32^c^	0.73

b values at ^a^0–800, ^b^0–1,000, ^c^0–1,500 and ^d^0–2,000 sec/mm^2^. ADC, apparent diffusion coefficient.
